# Trends in road accidents on Brazil’s highways: Evidence of persistence using fractional integration

**DOI:** 10.1371/journal.pone.0287302

**Published:** 2023-07-13

**Authors:** Peter Wanke, Luis Alberiko Gil-Alana, Yong Tan

**Affiliations:** 1 Federal University of Rio de Janeiro, Rio de Janeiro, Brazil; 2 University of Navarra, Pamplona, Spain; 3 Universidad Francisco de Vitoria, Madrid, Spain; 4 University of Bradford, Bradford, United Kingdom; Al Mansour University College-Baghdad-Iraq, IRAQ

## Abstract

This paper deals with the analysis of trends in road accidents on highways in Brazil. We use time series techniques based on fractional integration that allow us to determine if exogenous shocks in the data have transitory or permanent effects depending on the order of integration of the series. Our results indicate that a low degree of long memory was detected in the series with shocks having thus transitory effects over time. We further find that the number of accidents have been reducing over time, though in the presence of negative shocks, the recovery is not going to be immediate due to the long memory nature of the data. Despite the absence of relevant investment relating to infrastructure expansion, it is worth mentioning the consolidation of a nationwide tolled road system in Brazil involving concessions to private administrators, alongside more severe traffic laws that can impose limitations on driving licences.

## 1. Introduction

Road accidents are tragedies that lead to serious injuries and fatalities. Based on the report published by the World Health Organization (WHO) on the 21st June 2021 [1], the number of people who died from road traffic crashes reaches around 1.3 million every year. Looking at the statistics more specifically among countries with different income levels, the report found that low- and middle-income countries in the world account for 93% of the world’s fatalities caused by road accidents, although the total vehicles possessed by these countries only account for 60%. An ambiguous target has been set by the United Nations General Assembly to reduce the number of injuries and deaths caused by road accidents by half across the globe by the end of 2030.

The tremendous harm caused by road accidents is not only limited to the individual or the family level as reflected by the pain and loss derived from injuries or death, but its influence is extended further to a country level and in particular, it affects a country’s economy. Based on the report from the WHO, road accidents entailed a cost of 3% of gross domestic product for most countries in the world [1]. From the perspective of gross national product, Elvik [2] found that including an economic valuation of lost quality of life, the total cost of road accidents was estimated to be 2.5% of the gross national products.

Research in this area has been attracting the attention of the academic researchers and studies have investigated this issue from different perspectives due to the fact that it can potentially bring benefits to individuals, families and entire countries by reducing the number of accident blackspots where injuries or death occur. Indeed, a WHO report shows that among other types of diseases, the road accident is the major cause of death of children and young adults aged 5–29 years [1].

Looking at the previous studies in the related topic area, researchers have focused on different issues including the modelling of the relationship between weather and road accidents [3]; modelling road accident in the production process [4]; the connection between vehicle insurance and road accidents [5]; the link between vehicle load capacity and road accidents [6]; the effect of cell phones on vehicle accidents [7]; the influence of willingness-to-pay on road accidents [8]; among others.

It is not difficult to notice from the previous research studies that efforts have been made to investigate the road accident from the perspective of production economics by modelling the production process; also a great degree of concern has been focussed on investigating relevant factors which might reduce the volume of road accidents. Previously published studies contributed to both the empirical and theoretical perspectives, however, so far little attention has been made to look at the data in a more careful and thorough manner. More specifically, very few studies have looked at the pattern of road accidents by investigating persistence although this issue has been examined in other topic areas such as the persistence of urban air pollutant [9]; persistence of inflation [10]; persistence of efficiency [11] among others. One exception is the paper by Monedero et al. [12]. In that paper, the authors investigate road accidents in Spain using also a fractional integration approach. Their results indicate that the series examined display very low degrees of persistence, with the orders of integration being around 0 and thus showing a short memory pattern. The persistence of road accidents does not only affect the number of injuries and deaths in the long-term, but also hinders the management and control of road accidents.

Our study differentiates from Monedero et al. [12] by the fact that we investigate specifically on the Brazil’s highways rather than the road accidents in general. Narciso and Mello [13] argue that the injuries and death derived from the accidents on the highways is a global issue, especially in the developing countries such as Brazil. Therefore, it is worth of investigating the issue of road accident specially on the highways. However, the existing studies have not made enough attention on this issue. Andrade and Antunes [14] analyzed the trend in terms of the fatalities, major injuries as well as minor injuries derived from road accidents on Brazil’s federal highways before and after the start of the Decade of Action for Road Safety (DARS). They used the Prais-Winsten method to calculate the monthly percentage change in the number of fatalities, severe injuries and minor injuries. The findings suggest that there was an upward trend in terms of the number of victims and injuries before the introduction of DARS, while there was a downward trend afterwards. Another piece of study from Oliveira and Achcar [15], facilitated by Chi-square tests and logistic regression models, investigated the significant factors in the incidence of accidents on Brazil’s highways. The findings suggested that the serious injuries can be explained by several factors such as type of accident, day, highway type and vehicle type, while the dead victim depends on covariates age, time of day, highway type, highway facility, gender and type of vehicle. What we can conclude through reviewing the above studies on the investigations of accidents on Brazil’s highway is no effort has yet been made to use advanced time series techniques to look at the trend of accident on Brazil’s highway. Andrade and Antunes [14]’s method suffers from the limitation that simple calculation of monthly percentage change in the injuries and death lack of accuracy and robustness for the results. We contribute to the previous literature by providing a time series model to evaluate the persistence of road accidents using a data sample from Brazil. Thus, the main contribution of the present work is to analyze in detail the number of road accidents in Brazil’s highways, using updated time series techniques based on the concept of fractional integration. This technique is more flexible and general than the standard methods and permits us to evaluate the existence of trends in the data in a more consistent and efficient way. In other words, the novelty of the current study lies in the proposal and use of advanced statistical techniques (i.e. time series techniques based on fractional integration) to investigate the trend of accidents on the highways in Brazil, through which we would be able to obtain more robust estimates and simplify the estimation procedure. The proposal of this advanced estimation technique would provide a good example for future studies providing relevant statistical analysis in the similar topic area specifically and those providing trend analysis in general.

The current paper is structured as below: Section 2 presents the historical context, followed by Section 3 which outlines the innovative methodology adopted by the current study. Section 4 provides the data and the descriptive statistics. Section 5 presents and discusses the results. Finally, the concluding remarks are provided in Section 6.

## 2. Historical context

The World Health Organization (WHO) provides a number of indicators related to road crashes in most countries in the world, including Brazil. In particular, some of the data is related to the number of deaths in road traffic crashes provoked by alcohol-related factors. The data for Brazil shows that in 2016, 36.7% of male deaths in road accidents were attributed to alcohol related road traffic crashes, ranking 66^th^ in the world, while for females, 23% of deaths in road traffic crashes were connected to alcohol related factors, ranking 74^th^ in the world [16].

Not only does the WHO provide data regarding alcohol related deaths from road traffic crashes but relevant classifications have also been drawn up showing the distribution of road traffic deaths by type of road user. Based on the statistics for the year 2016, 23.2% of deaths involved drivers/passengers of 4-wheeled vehicles, 31.4% were from drivers/passengers of motorized 2- or 3- wheelers, deaths of pedestrians accounted for 18.1% with cyclists accounting for the lowest percentage, which was 3.4% [17]. Based on the data, it is recommended that priority should be given by the government and regulatory authorities to instigate policies to enhance safety for motorized 2–4 wheeled vehicles. According to a report from the World Bank global road safety facility, the population of Brazil was 207,652,864 in 2016, while the number of reported road crash fatalities in that year was 38,651 and the number of estimated serious injuries was 615,105. Logically, this resulted in substantial costs for the country. In fact, the reported cost reached $118,799 million, accounting for 6.6% of the country’s GDP in 2016 [18].

Based on the report from Brake (the Road Safety Charity), road accidents are the number one cause of death in the world for people aged 5–29 years old [19], while looking at the overall data for the whole population in Brazil. Table 1 shows the top 10 causes of death in Brazil and their world ranking. As we can see 20.18% of deaths are related to road traffic accidents in Brazil ranking 77^th^ in the world, following violence, lung disease and prostate cancer, the road accident related death is the fourth most serious social issue in Brazil.

**Table 1:** Top 10 causes of death in Brazil, their rate and ranking.

	Causes	Rate	World Ranking
1	Coronary Heart Disease	79.33	138
2	Stroke	51.38	122
3	Influenza and Pneumonia	42.68	76
4	Diabetes Mellitus	30.34	89
5	Violence	29.95	**10**
6	Lung Disease	28.67	61
7	Prostate Cancer	20.52	63
8	Road Traffic Accidents	20.18	77
9	Breast Cancer	15.78	97
10	Lung Cancers	13.93	87

We can also look at the death from traffic accidents by age group. The Ministry of Health of Brazil provided the statistics, which are shown in Table 2. As we can see from the table, the number of deaths from road accidents is mainly focused on three age groups, 20–29, 30–39 and 40–49 with the largest number of deaths corresponding to the first group. In comparison, the next three age groups are not particularly influenced by traffic accidents, these are 70 and over, 60–69 as well as 50–59, and they have the largest number of death derived from non-traffic accident causes.

**Table 2:** Deaths per age group.

Age Group	Deaths by traffic accident		Deaths by other causes	
	Number	%	Number	%
Less than 1	95	0.31	61846	6.44
1 to 9	1450	4.75	16096	1.68
10 to 19	3602	11.81	25691	2.67
20 to 29	7332	24.04	52104	5.42
30 to 39	6002	19.68	61901	6.44
40 to 49	4619	15.14	86987	9.06
50 to 59	3071	10.07	113311	11.80
60 to 69	2110	6.92	154776	16.11
70 and over	1934	6.34	383608	39.93
Ignored	286	0.94	4294	0.45
Total	30501	100	960614	100

Source: Ministry of Health

This section does not only serve as the purpose of providing background information about the issue of road accident in Brazil facilitated by relevant data, but also we would like to provide an overview regarding the research undertaken so far by the literature in this research topic. Looking through relevant studies published over the past decade (2012–2021), we observe that the studies on road accident can be classified into a few categories including a) road accidents forecast or prediction under different approaches [20, 21]; investigation on the determinants of road accidents [22, 23]; statistical analysis on the patterns, concentration and hotpots of road accidents [24–26], and estimation on the cost of road accidents [27];

Our additional observation related to the existing literature and on this topic in different areas of Research, as illustrated above experiences an uneven development in terms of the number of studies included and the depth of analysis employed. In particular, it is noticed that the majority of previous research works focused on the investigation of road accidents determinants, with significantly less attention paid to road accidents data in a careful and thorough manner. More specifically, there are very limited studies concentrating on the examination of the road accident patterns, in particular under advanced statistical times series techniques. In the context of Brazil’s transportation industry and in particular, the area of road accident on Brazil highways, as far as we are concerned, no effort has yet been made to investigate the patterns of accidents using advanced techniques. We are motivated by this fact the most importantly, the significance of our study lies to the fact that we would be able to provide useful policies to the urban regulatory planner for their regulation on the transportation industry to reduce accidents.

## 3. Methodology

There are many modelling approaches to deal with persistence in time series data. The most standard model is to consider an autoregressive of order 1, i.e., AR(1) process of form:

$$(1-\alpha L)x_{t}=u_{t},\;t=1,2,\ldots ,$$ (1)

where x_t_ refers to the observed data, L is the lag-operator (L^k^ = x_t-k_), α is the AR coefficient and where u_t_ is a white noise process. In this context, the parameter α measures the degree of persistence, and if α = 1, x_t_ is said to contain a unit root and be nonstationary, with shocks having permanent effects on the evolution of the series. If α < 1, x_t_ is stationary and shocks will be transitory, being the effect shorter as lower the value of α is.

The model can be generalized to an AR(p) process of the form:

$$\phi_{p}(L)x_{t}=u_{t},\;t=1,2,\ldots ,$$ (2)

where ϕ_p_(L) = (1 - ϕ_1_L - …—ϕ_p_ L^p^), and persistence is measured then as the sum of the AR coefficients, i.e., ϕ = (1 + ϕ_1_ + … + ϕ_p_).

A problem with these autoregressive approaches is that they produce an abrupt change in the behaviour of the series as α (or ϕ) approaches unity, moving from the stationary to the nonstationary case. This is solved by using alternatives based on fractional integration, which use the following equation,

$${\left(1-L\right)}^{d}x_{t}=u_{t},\;t=1,2,\ldots ,$$ (3)

and where d can be any real value. Note, that for any real value of d, the polynomial in L in the left hand side of Eq (3) can be expanded as:

$${\left(1-L\right)}^{d}=\sum_{j=0}^{\infty }\left(\begin{array}{l} d\\ j \end{array}\right){\left(-1\right)}^{j}L^{j}=1-dL+\frac{d(d-1)}{2}L^{2}$$ (4)

and thus, x_t_ in (3) depends on all its past history if d is a fractional value. Moreover, and based on the above equation, the higher the value of d is, the higher the dependence between the observations is, and thus, d can be considered as an indicator of the degree of persistence in the data. Note that in this context, the unit root nonstationary model corresponds to d = 1 in (3) but there is no an abrupt change in its behaviour around 1. Using this fractional approach, shocks will be transitory if d is strictly smaller than 1 and lower the value of d is, the faster is the convergence process to its long term evolution. On the contrary, values of d equal to or higher than 1 imply lack of mean reversion with shocks having permanent effects. 1 The concept of fractional integration was introduced by Granger [28, 29], Granger and Joyeux [30] and Hosking [31]. Granger [28] realized that many series that were nonstationary and that apparently required first differentiation, once being first differenced appeared to be overdifferenced, suggesting the need of fractional degrees of differentiation.

In addition, and based on the standard approaches in the unit root literature [32, 33] we permit x_t_ to be the errors in a regression model that incorporates a linear time trend of the form:

$$y_{t}=\alpha +\beta t+x_{t},\;t=1,2,\ldots ,$$ (5)

where y_t_ are the original time series data.

We estimate the order of integration of the series, d, using a frequency domain version of the Whittle function [34] by using a testing procedure developed in Robinson [35] that is particularly appropriate with our data compared with other methods. Thus, it behaves well in finite samples [36] which is important in the present work given the limited number of observations used in this application. Moreover, the method remains valid even in nonstationary contexts (i.e., d ≥ 0.5) unlike most estimating/testing procedures where stationarity is a pre-requisite; thus, using the method of Robinson [35] we do need the requirement of preliminary differencing in the case of nonstationary series. Finally, it has a standard null limit distribution (unlike most unit roots methods that rely on critical values calculated case by case in simulation studies), and it is the most efficient method in the Pitman sense against local departures from the null [35].

## 4. Data and descriptive statistics

The Brazilian road accident data were obtained from the ANTT Dados Abertos website (https://dados.antt.gov.br/dataset/acidentes-quilometro-rodovias), available on a daily basis from 2010 to 2021. Since the original downloaded dataset indicates, per road, every km that had an accident in a given day, and we had to aggregate this information to obtain how many accidents occurred per day on each road, as displayed in Table 3, in major tolled Brazilian roads, namely: (i) Fernão Dias (links the cities of São Paulo and Belo Horizonte); (ii) Autopista Fluminense (links the cities of Rio de Janeiro and Campos, the major oil offshore production pole in Brazil); (iii) Litoral Sul (links major cities in the Southern parts of Brazil, such as Curitiba and Florianópolis); (iv) Planalto Sul (also links major cities in the Southern parts of Brazil, such as Curitiba and Florianópolis, but stretches down to the borders with Argentina in Rio Grande do Sul state); and (v) Regis Bittencourt, also known as the “road of death”, which links the cities of São Paulo and Curitiba.

**Table 3:** Descriptive statistics for the number of accidents per day in major Brazilian roads.

Descriptive Statistics	Fernão Dias	Fluminense	Litoral Sul	Planalto Sul	Regis Bittencourt
Min	1	0	1	0	0
Median	20	9	25	5	13
Max	109	36	128	20	96
Mean	23.38	9.97	26.32	5.22	15.63
SD	12.67	4.52	10.78	3.00	10.29
CV	0.54	0.45	0.41	0.57	0.66
Skewness	1.92	0.84	1.43	0.72	2.12
Kurtosis	5.63	1.36	4.82	0.78	7.00
Information Entropy	0.43	0.34	0.41	0.29	0.41

● SD stands for standard deviation and CV represents coefficient of variation.

**Fig 1:**
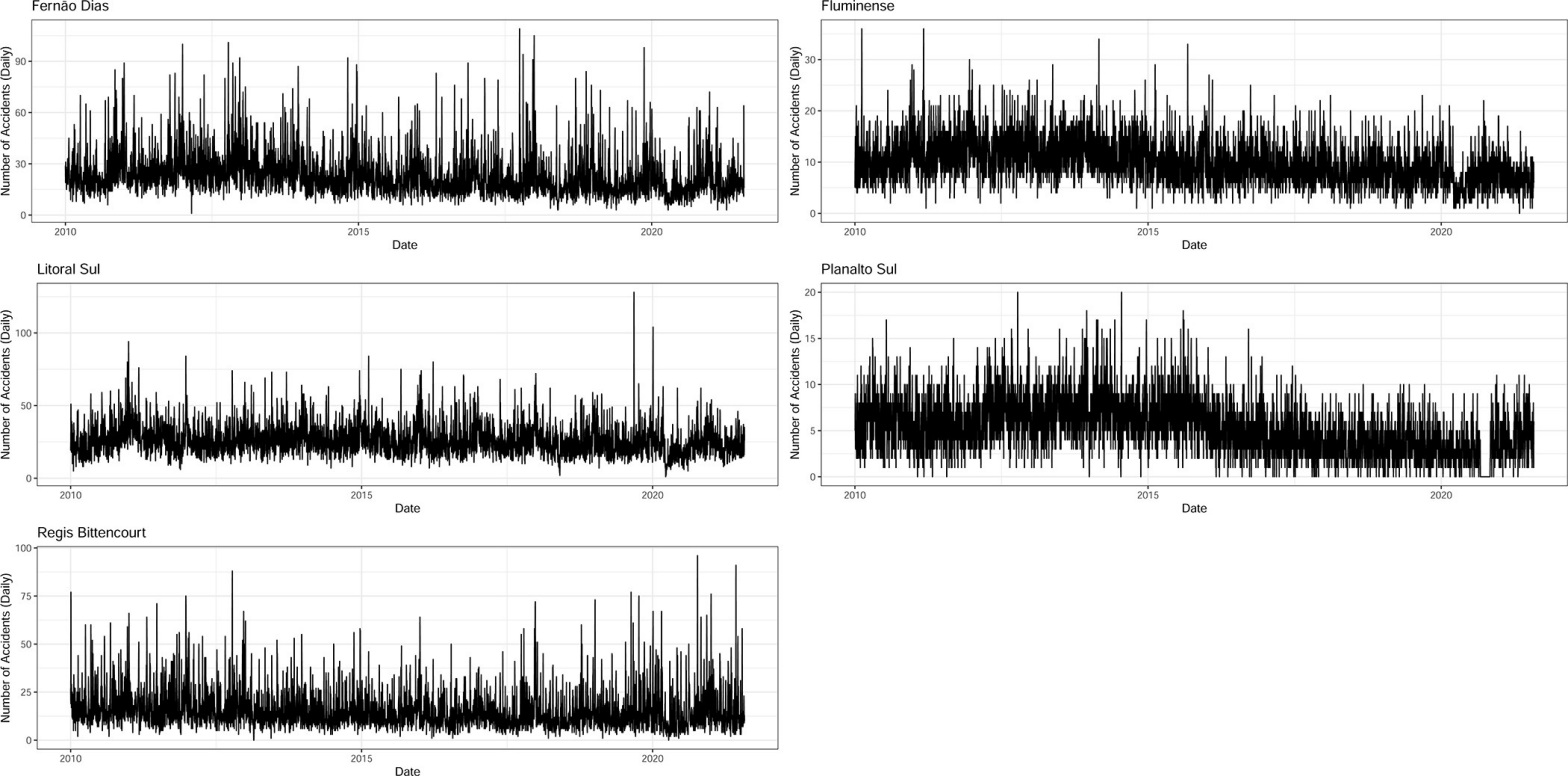
Number of accidents per road (daily).

**Fig 2:**
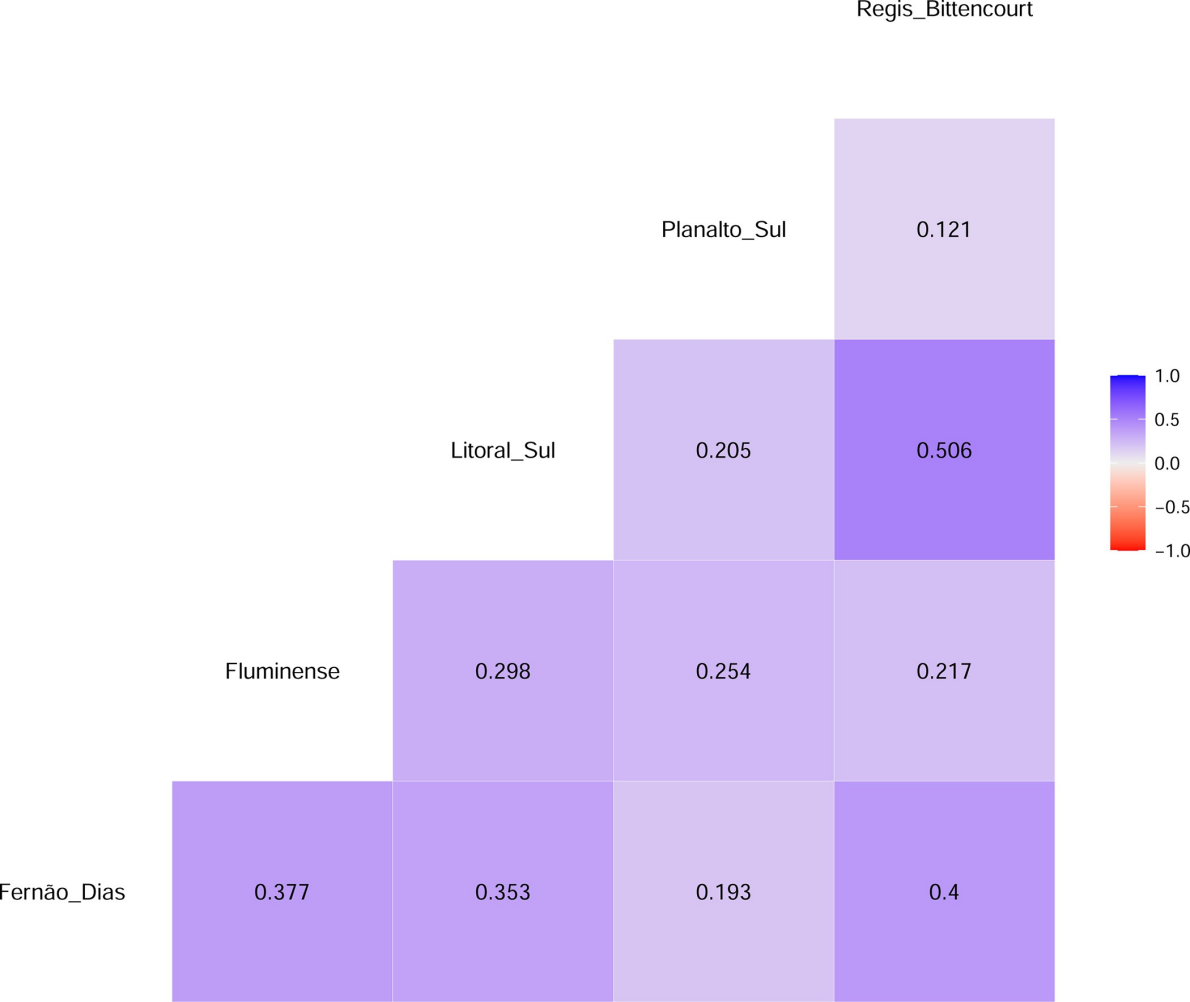
Correlogram of roads accidents.

Fig 1 displays the time series plots and suggests a strong seasonal pattern of daily accidents in major Brazilian roads, mostly driven by effects such as weekdays and holidays, no significant increasing or decreasing trend can be captured by a mere glimpse. Besides, daily accidents between these roads seem to be uncorrelated (cf. Fig 2), confirming these roads are independent logistics systems. We can see from Table 1 that there is a large difference in terms of the maximum number of accidents that took place on the roads with very tiny difference noticed in terms of the minimum number of accidents, this also results in a large difference in the standard deviation. In summary, the statistics show that the maximum daily number of accidents took place in Litoral Sul, Fernão Dias and Regis Bittencourt, these three roads experiencing the largest standard deviation. With regard to skewness, we notice that Fernão Dias, Litoral Sul, and Regis Bittencourt were highly skewed in the distributions, with the distributions of the remaining two roads being moderately skewed. The distributions of three roads, namely Fernão Dias, Litoral Sul and Regis Bittencourt are leptokurtic, while the distributions of the other two roads were found to be platykurtic. Finally, it was found that all the roads in the sample exhibit a degree of disorder in the data, although Planalto Sul performed the best.

## 5. Empirical results

We consider the model given by the Eqs (3) and (5), i.e.,

$$y_{t}=\alpha +\beta t+x_{t},\;{\left(1-L\right)}^{d}x_{t}=u_{t},\;t=1,2,\ldots ,$$ (6)

where y_t_ is the time series we observe, α and β are unknown coefficients referring respectively to a constant and a time trend, and x_t_ is supposed to be I(d) where the order of integration d is an additional parameter to be estimated from the data; finally, u_t_ is an error term that is supposed to be uncorrelated with zero mean and constant variance. Remember that the parameter d indicates the degree of persistence and it determines if exogenous shocks in the data will have permanent or transitory effects.

### 5i) Analysis of the results

Table 4 reports the estimated values of the differencing parameter d in (6) under the three classical assumptions in the unit roots literature: i) no deterministic terms, i.e., imposing that α = β = 0 a priori in (6); ii) with an intercept (only β = 0 a priori), and iii) with an intercept and a linear time trend. If these two coefficients are significant we keep this model, otherwise we move to the model with an intercept, and if this is also insignificant, to the case with no terms.

**Table 4:** Estimates of the differencing parameter, d, and 95% confidence intervals.

Series	No terms	With a constant	With a constant and a linear time trend
Fernão Dias	0.17 (0.15, 0.19)	0.11 (0.09, 0.13)	**0.06 (0.04, 0.08)**
Fluminense	0.21 (0.19, 0.22)	0.14 (0.13, 0.16)	**0.05 (0.04, 0.07)**
Litoral Sul	0.15 (0.12, 0.17)	0.07 (0.05, 0.08)	**0.03 (0.01, 0.05)**
Planalto Sul	0.22 (0.20, 0.23)	0.17 (0.16, 0.19)	**0.12 (0.11, 0.14)**
Regis Bittencourt	0.11 (0.09, 0.13)	0.06 (0.04, 0.08)	**0.04 (0.02, 0.06)**

The values in parenthesis are the 95% confidence interval for the values of d. In bold, the selected model in relation with the deterministic terms.

We have marked in bold in Table 4 the significant case for each series, and we notice that the time trend is statistically significantly negative in all five series, implying a constant decrease in the long run evolution of the series. Focussing on the differencing parameter, we observe that the estimates of d are all significantly positive though very close to 0.

### 5ii) Discussion of the results

The results indicate that though long memory is detected (d > 0), the values are very small, with shocks disappearing extremely fast. Table 5 focuses on the estimated coefficients of these selected models, and we observe that the time trend is negative in all cases, ranging their values from -0.00094 (Planatol_Sul) to -0.00259 (Fernao_Dias). These negative trends indicate that the number of accidents is reducing over time; however, the fact that d is positive indicates the presence of a long memory pattern, so negative shocks in the series (for instance, an increasing number of accidents) disappear relatively slowly (or at least slower than under the classical case of d = 0 rejected in our model), requiring therefore some action to return to their original long term projections.

**Table 5:** Estimated coefficients in the selected models.

Series	D	Intercept (tvalue)	Time trend (tvalue)
Fernão Dias	0.06 (0.04, 0.08)	28.624 (50.28)	-0.00259 (-10.90)
Fluminense	0.05 (0.04, 0.07)	12.887 (71.35)	-0.00139 (-19.11)
Litoral Sul	0.03 (0.01, 0.05)	29.069 (72.15)	-0.00131 (-8.04)
Planalto Sul	0.12 (0.11, 0.14)	7.173 (38.83)	-0.00094 (-12.87)
Regis Bittencourt	0.04 (0.02, 0.06)	17.876 (43.26)	-0.00104 (6.27)

## 6. Conclusion

In this article we have examined the number of road accidents in the main roads in Brazil by using time series techniques based on fractional integration. Our study significantly contributes to the literature on the analysis of road accident in general and the examination of the road accident patterns on Brazil highways specifically by providing an advanced time series statistical technique in the analysis. According to our results, the five series examined display significant negative time trends, implying a reduction in the number of accidents over time; however, at the same time the series display long memory, a feature that indicates that shocks tend to be persistent and have long-lived effects. This long memory pattern, however, is small since the coefficient, though statistically different from zero, it is relatively very close to it.

Comparing the coefficents of the time trends between the I(0) specification for the error term and the I(d) approach, we notice, in Table 6 that the size of the coefficients for all the roads are smaller under the long memory approach implying that the reduction in the number of accidents is smaller than the number provided by the short memory or I(0) approach. Nevertheless, the differences are small. The differences between short and long memory approaches can be explained by the current status of infrastructure expansion investments, the granting of current road infrastructure to private concessionaires, and more rigid legal apparatus and the introduction of more severe traffic laws to punish negligent drivers. While road infrastructure in Brazil has remained stagnant over the last four decades, conjunctural improvements emerged in terms of a nationwide toll system capable of maintaining the current road infrastructure minimally operational and safe. On the other hand, civil society pressed congressmen and authorities to approve a traffic law similar to the one ratified in EU countries, where negligent drivers can lose their driving licence after a number of incidents. Most of this pressure emerged as a consequence of the higher fatality rates in Brazilian roads, even when compared to other countries and diseases.

**Table 6:** Time trend coefficients under I(0) and I(d) errors.

Series	d = 0	d estimated
Fernão Dias	-0.00265	-0.00259
Fluminense	-0.00141	-0.00139
Litoral Sul	-0.00132	-0.00131
Planalto Sul	-0.00099	-0.00094
Regis Bittencourt	-0.00105	-0.00104

This article can be extended in several directions. For example, as a robustness method, the long memory methodology used in this work can be extended to other parametric or even non-parametric methods. In addition, the presence of structural breaks can also be taken into account and a comparison of these results with other smaller roads or even other countries are issues which are currently in progress. In the same line, the linear trends investigated in this work can be replaced by non-linear ones by using for example Chebyshev polynomials in time as in Cuestas and Gil-Alana [37] or Fourier functions [38] or even neural networks [39] still within the context of fractional integration. Finally, although the current study benefits from the advantage and ability to look at and analyze the data in a great detail from the proposed method, our work, on the other hand, suffers from the issue that only the road accident data is included, which is different from the existing literature which investigated the determinants for the incidence of road accident or the relationship between road accident and other variables concerned. Therefore, another area of future studies would be looking at the road accidents data while simultaneously consider other variables as well, from this perspective, the future study can benefit from the ability to compare the results with a wide range of other existing studies.

## Supporting information

S1 Data (XLSX)
